# Role of metabolic conditions in cardiorenal diseases: initiating pathways and therapeutic targeting

**DOI:** 10.3389/fnut.2025.1701084

**Published:** 2025-11-19

**Authors:** Yeshun Wu, Hongqing Xu, Xiaoming Tu, Zhenyan Gao

**Affiliations:** Department of Cardiology, The Quzhou Affiliated Hospital of Wenzhou Medical University, Quzhou People’s Hospital, Quzhou, China

**Keywords:** metabolic conditions, albuminuria, kidney injury, cardiovascular outcomes, mechanism, intervention strategies

## Abstract

Albuminuria, a core indicator of kidney injury, is closely associated with cardiovascular disease prognosis. Through multiple mechanisms, metabolic abnormalities, such as hyperglycemia, hyperuricemia, obesity, and dyslipidemia, contribute to the onset and progression of albuminuria and significantly increase the risk of adverse cardiovascular outcomes. Based on recent clinical studies and basic experimental evidence, this review systematically elucidates how metabolic conditions are involved in the relationship between albuminuria and cardiac prognosis, encompassing several mechanisms, including chronic inflammation, endoplasmic reticulum stress, renin–angiotensin–aldosterone system overactivation, hemodynamic alterations, vascular endothelial dysfunction, mitochondrial dysfunction, and lipotoxicity. Additionally, it explores clinical intervention strategies. This review underscores the pivotal role of metabolic conditions in driving cardiorenal diseases and outlines evidence-based strategies for clinical management.

## 1 Introduction

**Figure 1:**
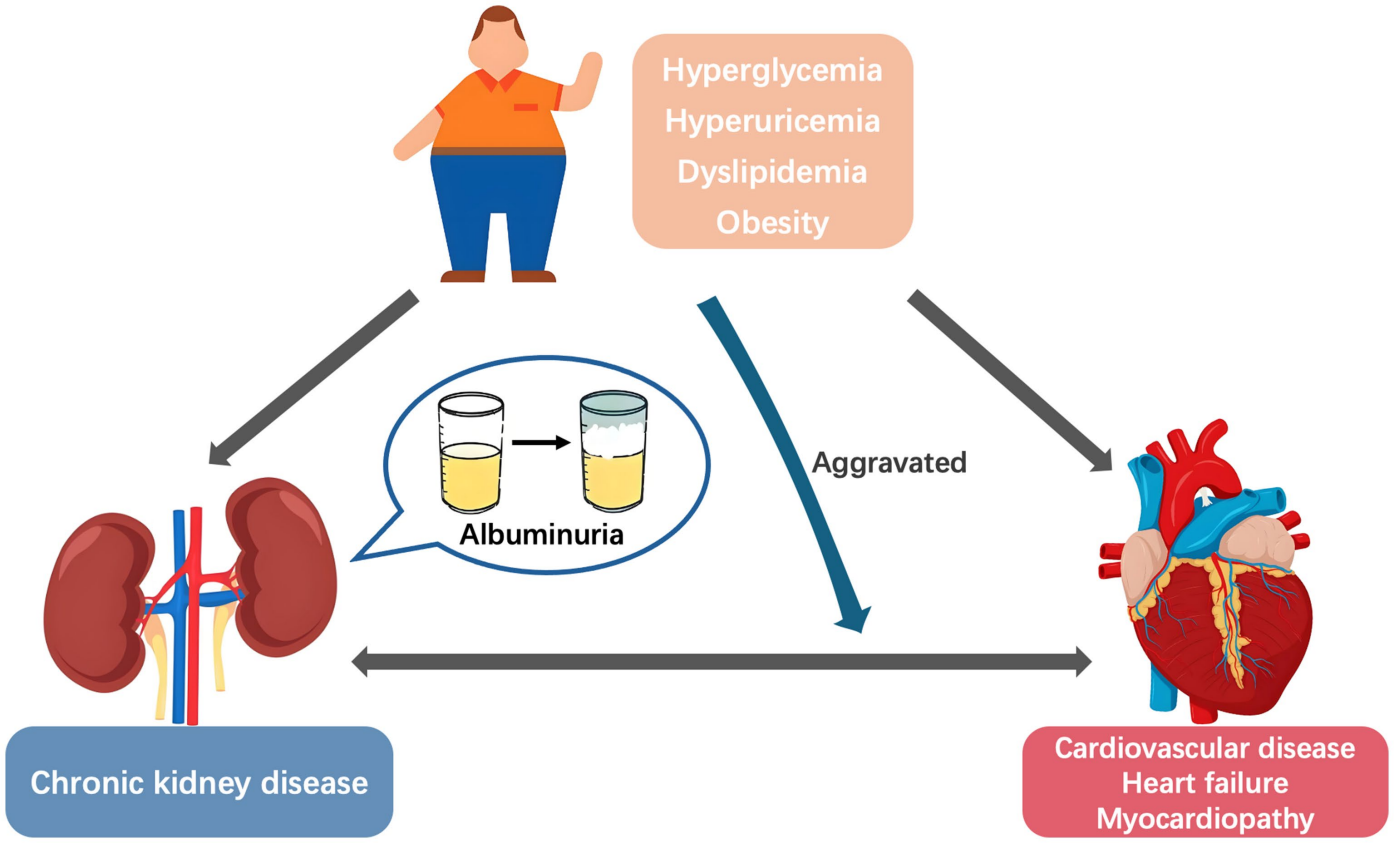
The vicious “metabolic abnormalities–albuminuria–cardiovascular disease” cycle.

Albuminuria is a core indicator of kidney disease progression and an independent risk factor for cardiovascular disease (CVD) and heart failure (HF) (1–3). With the rising incidence of metabolic diseases, the role of metabolic conditions in the relationship between albuminuria and adverse cardiovascular outcomes has gained increasing attention. Metabolic disorder, renal disease, and CVD often overlap and coexist in affected individuals. In a study involving 11,607 American adults, approximately 26.3% had at least one cardiac, renal, or metabolic disease, 8.0% had two of these conditions, and 1.5% had all three diseases simultaneously (4). Moreover, metabolic risk factors were the main CVD-attributable burdens in China, increasing from 62.80% in 1990 to 70.45% in 2019 (5). Common metabolic abnormalities, including hyperglycemia, hyperuricemia, dyslipidemia, and obesity, not only share common pathophysiological mechanisms with cardiorenal diseases but also exacerbate disease progression when coexisting (6, 7). This interrelationship has led to the concept of Cardiovascular–Kidney–Metabolic syndrome (8, 9). However, metabolic abnormalities appear to be the primary driver, rather than mere contributors, of the “metabolic abnormalities–albuminuria–CVD” cycle. Metabolic abnormalities trigger albuminuria through kidney injury (10) while directly promoting vascular damage (11, 12), thereby initiating the progression of cardiorenal disease (Figure 1). Based on recent clinical research and basic experimental evidence, this research aimed to systematically elucidate the role of metabolic conditions as a driving factor in the relationship between albuminuria and cardiovascular prognosis, and explores the clinical intervention strategies.

## 2 Correlation between albuminuria and cardiac outcomes

Previously, a 24-h albumin excretion rate (AER) exceeding 30 mg was the gold standard for diagnosing albuminuria (13). However, given that 24-h urine collection is often impractical and causes patient burden, spot urine samples are now routinely used to estimate AER clinically (13). Assuming approximately 1 g of urinary creatinine is excreted daily, the urinary albumin-to-creatinine ratio (UACR), calculated using spot urine samples, is used to determine the presence of albuminuria (14). Given that UACR remains relatively constant and is not influenced by changes in individual urine volume or body weight, it features high accuracy and reliability (14).

Currently, albuminuria, defined by the criterion of UACR >30 mg/g, is recognized as a critical indicator closely associated with the progression and adverse outcomes of chronic kidney disease (CKD), particularly diabetic nephropathy (DN) (15). Mounting epidemiological evidence suggests that urinary albumin excretion is also linked to CVD incidence and mortality, with albuminuria being an independent risk factor for cardiovascular events (16, 17). A prospective cohort study examined 8,975 patients with type 2 diabetes mellitus (T2DM) without pre-existing CVD at baseline and found that, after a median follow-up of 4.05 years and adjustment for potential confounders, participants with microalbuminuria exhibited a higher CVD risk, with a hazard ratio (HR) of 1.57 (95% confidence interval [CI]: 1.04–2.37) for myocardial infarction (MI) and 1.30 (95% CI: 1.07–1.57) for total CVD. Moreover, as the UACR increased, the risk also increased. Those with macroalbuminuria had an HR of 2.86 (95% CI: 1.63–5.00) for MI and 2.42 (95% CI: 1.85–3.15) for total CVD (18).

Traditionally, individuals who do not meet the diagnostic criteria for CKD (UACR < 30 mg/g, estimated glomerular filtration rate [eGFR] > 60 mL/min/1.73 m^2^) are not considered to have high CVD risk. Nevertheless, recent retrospective clinical studies have presented contradictory findings, revealing a correlation between elevated UACR within the normal range and cardiorenal risk, independent of eGFR levels (19, 20). Research utilizing data from the National Health and Nutrition Examination Survey demonstrated a near-linear relationship between continuous UACR levels and CVD risk, even among individuals without apparent cardiovascular disease, underscoring the continuum of risk and the importance of early intervention (19). Similarly, after adjusting for sociodemographic information, body mass index (BMI), smoking status, baseline eGFR, and related comorbidities, Kang et al. found that a UACR within 6.211–10.010 mg/g was already significantly associated with increased cardiac mortality (HR = 1.51, 95% CI: 1.12–2.03, *p* = 0.006). This association further intensified when UACR exceeded 10.010 mg/g (HR = 2.14, 95% CI: 1.62–2.82, *p* < 0.001) (21).

Chronic kidney injury reportedly elevates cardiovascular risk through multiple pathophysiological processes, including endothelial dysfunction, diffuse vascular damage, systemic inflammation, atherosclerosis, myocardial remodeling, and sodium and water retention (22–24). Interestingly, through Mendelian randomization analysis, Zhou et al. discovered that elevated UACR exhibited a causal relationship with increased risks for CAD (odds ratio [OR], 1.260; 95% CI: 1.042–1.523; *p* = 0.017) and MI (OR, 1.424; 95% CI: 1.137–1.783; *p* = 0.002). However, this causal relationship vanished after adjusting for metabolic factors such as blood pressure, blood glucose, and lipid levels (25), suggesting that the detrimental impact of UACR on CAD is mediated by these metabolic conditions.

## 3 Role of metabolic conditions in cardiorenal prognosis

Metabolic conditions such as hyperglycemia, dyslipidemia, obesity, and hyperuricemia are well-established independent risk factors for albuminuria, driving its progression from microalbuminuria to macroalbuminuria and from intermittent to persistent states.

A study by Sivanantham et al. demonstrated that microalbuminuria incidence was 27.7% (95% CI: 18.1–38.6) among patients with hypertension alone and 40.6% (95% CI: 29–52.2) among those with diabetes alone (26). In a survey conducted across 105 primary care units in Turkey, diabetes was significantly associated with an increased albuminuria risk (OR, 1.667; 95% CI: 1.205–2.309; *p* = 0.002). Moreover, albuminuria prevalence was significantly lower in patients with diabetes with controlled blood glucose than in those without controlled blood glucose (59.0% vs. 47.0%, *p* = 0.002) (27). Notably, when combined with metabolic abnormalities, the correlation between albuminuria and cardiovascular prognosis changes. A multicenter registry cohort study involving 5,960 patients with CAD demonstrated that an increased UACR had a more significant impact on all-cause and cardiovascular mortality in patients with T2DM than in those without T2DM. Furthermore, an interaction between glycemic status and UACR levels was observed in relation to cardiovascular and all-cause mortality (both interaction *p*-values < 0.001), even when UACR values were within the guideline-recommended normal range (23).

Regarding the relationship between hyperlipidemia and albuminuria, Hwang et al. found that UACR was positively correlated with total cholesterol and triglyceride levels but negatively correlated with high-density lipoprotein cholesterol (HDL-C) levels (28). Similarly, a higher ratio of non-HDL-C to HDL-C was significantly associated with an increased risk of macroalbuminuria (OR, 1.34; 95% CI: 1.13–1.59; *p* = 0.0007). A subgroup analysis revealed that this association was stronger among participants with a BMI of ≥30 kg/m^2^ (OR, 1.89; 95% CI: 1.44–2.47; *p* < 0.01), even after excluding those taking medications that affect lipid metabolism (29). Moreover, as BMI, waist circumference, and body fat content gradually increase, urinary albumin excretion also increases (30). In genetic studies, HindIII polymorphism in the *LPL* gene, a key enzyme in triglyceride metabolism, is significantly associated with increased microalbuminuria risk in patients with T2DM (31). Moreover, Shao et al. quantitatively analyzed HDL proteome alterations using isotope dilution tandem mass spectrometry and found that low concentrations of the anti-atherosclerotic protein PON1 in the HDL proteome were associated with albuminuria and coronary artery calcification. In patients with T1DM manifesting albuminuria, reduced PON1 levels in the HDL proteome may partially mediate the increased CVD risk, increasing the possibility that HDL proteome alterations act as mediators of kidney disease and atherosclerosis risk (32).

A community-based prospective cohort study involving 1,862 middle-aged and older adult participants found that, over a 4-year follow-up period, after adjusting for confounding factors, each 1 mg/dL increase in serum uric acid (UA) was associated with a 1.42-fold higher risk of developing microalbuminuria (OR, 1.42; 95% CI: 1.27–1.59; *p* < 0.01). Thus, elevated serum UA can independently predict the onset of microalbuminuria (33). Additionally, Russo et al. conducted a retrospective investigation involving 21,963 patients from the URRAH study database. During a follow-up period of 215,618 person-years, they found that cardiovascular mortality stratified by all levels of eGFR was significantly higher in patients with hyperuricemia and proteinuria than in those with only one risk factor or no risk factors (34).

## 4 Mechanisms of metabolic conditions involved in cardiorenal diseases

**Figure 2:**
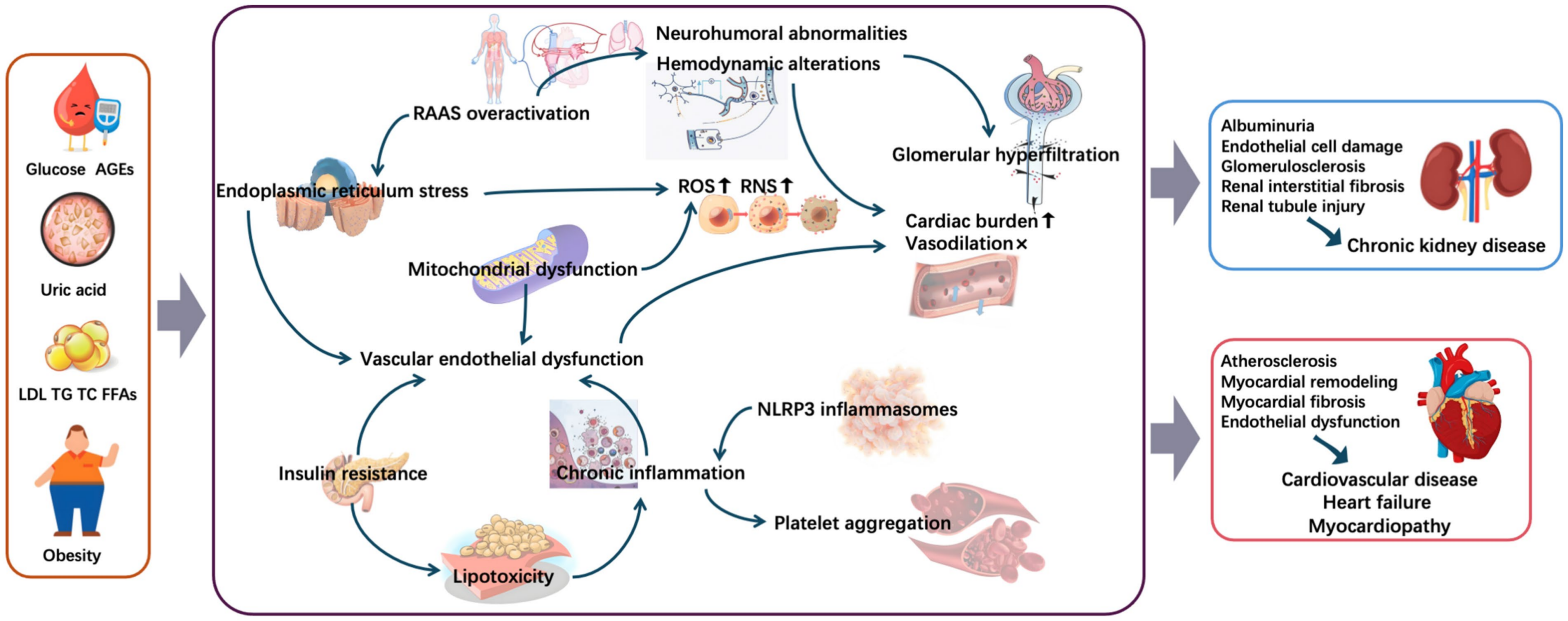
Mechanisms underlying metabolic conditions involved in cardiorenal diseases. AGEs, advanced glycation end-products; FFAs, free fatty acids; LDL, low-density lipoprotein; NLRP3, nodular receptor protein 3; RAAS, renin–angiotensin–aldosterone system; RNS, reactive nitrogen species; ROS, reactive oxygen species; TC, total cholesterol; TG, triglyceride.

Metabolic abnormalities deliver a “double hit” through renal injury (triggering albuminuria) and vascular damage (directly promoting atherosclerosis), creating a vicious “metabolic abnormalities–albuminuria–CVD” cycle. This multidimensional effect dominated by metabolic abnormalities may include the following specific underlying mechanisms (Figure 2).

### 4.1 Chronic inflammation and endoplasmic reticulum stress

Chronic low-grade inflammation induced by metabolic disorders exists in multiple organs or tissues, including the heart, brain, kidneys, and skeletal muscles and is characterized by the infiltration of immune cells, production of abnormal cytokines, and aberrant activation of inflammatory signaling pathways (35–38). Chronic inflammation is a fundamental feature of most renal pathologies, where inflammatory cell infiltration into the renal interstitium promotes fibroblast proliferation and collagen synthesis, causing renal interstitial fibrosis—a crucial process leading CKD to end-stage renal disease (39–41). Moreover, metabolically mediated inflammation could accelerate glomerulosclerosis and interstitial fibrosis, causing gradual decline in renal function (40). Additionally, inflammation may compromise the structural and functional integrity of glomerular cells and injure renal tubular epithelial cells, thereby impairing reabsorption and secretion functions and accelerating the progression of CKD (42–44). In populations with diabetes and obesity, elevated glucose and lipid accumulation can promote inflammatory responses dependent of nodular receptor protein 3 (NLRP3) inflammasomes, inducing podocyte injury, a major factor in subsequent renal damage (45–49). Furthermore, inflammation with a high-specificity cellular and molecular response contributes to the initiation and progression of atherosclerosis (50). A high-fat diet can exacerbate atherosclerosis by elevating the neutrophil levels (51). Chronic inflammation associated with metabolic disorders can also damage vascular endothelial cells and promote lipid deposition and platelet aggregation, ultimately forming atherosclerotic plaques and increasing CAD risk (52, 53). In summary, chronic inflammation driven by metabolic dysregulation may be a key contributor to the development of cardiorenal diseases associated with metabolic abnormalities.

Several studies have demonstrated that excess nutrients and inflammatory cytokines associated with metabolic diseases can trigger or exacerbate ER stress and induce the overproduction of downstream reactive oxygen species (ROS) and reactive nitrogen (54–57). ER stress disrupts the balance between nitric oxide and ROS, leading to oxidative stress, which aids in inducing endothelial dysfunction and atherosclerosis (58–60). Disruption of redox homeostasis leads to the accumulation of oxidative intermediates, which then attack unsaturated fatty acids in biological membranes, trigger lipid peroxidation, and further decompose into smaller oxides, including malondialdehyde; consequently, a series of structural and functional abnormalities involving the cardiovascular and renal systems occur (61–65). Thus, metabolically related ER stress facilitates the development of cardiorenal complications.

### 4.2 Overactivation of the renin–angiotensin–aldosterone system

During obesity, adipose tissue secretes various adipokines (e.g., leptin and adiponectin), which can directly or indirectly activate the renin–angiotensin–aldosterone system (RAAS) (66, 67). RAAS can also be activated by chronic hyperglycemia and hyperuricemia through the direct stimulation of renal renin secretion (68–71). This system is involved in blood pressure regulation, fluid homeostasis, and electrolyte balance. When it is overactivated, aldosterone secretion increases, promoting the renal tubular reabsorption of sodium and water, increasing volume load, and exacerbating cardiac burden (72). Angiotensin II induces plaque formation during the early stages, representing one of the most crucial impacts on atherogenesis from the RAAS (73). Additionally, the increased production of angiotensin II induces endothelin expression, which causes systemic vasoconstriction, blood pressure elevation, and cardiac and renal injury worsening (74, 75). Meanwhile, angiotensin II overexpression can induce oxidative stress, ER damage, and apoptosis by activating signaling pathways such as the mTOR/ERK pathway, leading to cardiorenal organ remodeling and dysfunction (76–78). Moreover, UA and angiotensin II synergistically increased inflammation and oxidative stress in human proximal tubular cells through the activation of toll-like receptor 4 (TLR4), in an additive manner (79). In summary, RAAS overactivation associated with metabolic abnormalities induces a vicious cycle of neurohumoral abnormalities, internal environment disturbances, and oxidative stress, further contributing in the development of cardiorenal disease.

### 4.3 Hemodynamic alterations

Obesity exhibits an increase in circulating blood volume and glomerular pressure, resulting in mechanical damage to the capillary walls. Prolonged exposure to elevated intraglomerular pressure can induce focal segmental glomerulosclerosis, which clinically presents as albuminuria and progressive decline in renal function (80, 81). In hyperglycemia, the secretion of vasoactive substances such as prostaglandins and nitric oxide increases, causing the afferent arterioles to excessively dilate and the renal blood flow to significantly increase, resulting in hyperfiltration (82, 83). Obesity and hyperglycemia promote the generation of advanced glycation end-products (AGEs), which are deposited in the glomerular basement membrane and mesangial region; this deposition leads to the thickening of basement membranes, proliferation of mesangial matrix, and restricted dilation of the efferent arterioles, exacerbating the hyperfiltration state (84–86). Prolonged hyperfiltration mechanically damages the glomerular capillary walls and continuously activates mesangial and endothelial cells, which release profibrotic factors such as TGF-β and platelet-derived growth factor, ultimately promoting glomerulosclerosis (87–91). At the macro level, reciprocal communication between the renal microvasculature and the systemic circulation creates a vicious cycle that accelerates the progression of cardiorenal disease (92). Additionally, hemodynamic disturbances associated with metabolic dysfunction increase cardiac load and exacerbate HF symptoms (93).

### 4.4 Vascular endothelial dysfunction

Metabolic abnormalities, such as hyperglycemia, hyperuricemia, and excessive release of free fatty acids (FFAs), adversely affect the vascular wall, leading to endothelial dysfunction (94–98). Vascular endothelial cells help maintain the barrier function between blood and the vascular wall; they also regulate the normal functioning of the circulatory system by balancing vasodilation and vasoconstriction (99). Vascular endothelial dysfunction is characterized by impaired endothelium-dependent vasodilation, increased oxidative stress, chronic inflammation, increased permeability, and endothelial cell senescence, collectively hindering the physiological and protective functions of endothelial cells (100, 101). In endothelial dysfunction, vascular regulatory mechanisms are impaired, increasing the risk of cardiorenal diseases. Such dysfunction also leads to the decreased activity of endothelial nitric oxide synthase, resulting in reduced NO synthesis, which enhances vasoconstrictive capacity and increases peripheral resistance, thereby exacerbating cardiac load and causing renal diseases (102–105). High levels of glucose and UA, as well as AGEs, increase the permeability of glomerular endothelial cells, induce endothelial cell apoptosis, significantly alter the glomerular filtration barrier, and lead to albuminuria (84, 106). Therefore, improving endothelial function may help prevent or treat cardiorenal diseases associated with metabolic abnormalities.

### 4.5 Mitochondrial dysfunction

Mitochondrial quality control is a core regulatory system that maintains cellular energy homeostasis through three synergistic mechanisms (107). First, PGC-1α–mediated mitochondrial biogenesis continuously generates new mitochondria by activating the protein nuclear factor erythroid 2–related factor 2. Second, mitochondrial dynamics—coordinately regulated by fusion proteins including mitofusins and fission proteins such as DRP1, ensure the clearance of damaged organelles and the renewal of healthy mitochondria with minimal resources and energy required. Finally, mitophagy mediated by PINK1/Parkin pathway, precisely identifies and eliminates damaged units with dissipated membrane potentials, thereby establishing a closed-loop system for mitochondrial quality control. This dynamic cycle—comprising biogenesis, remodeling, and clearance—provides adaptive support for cellular metabolism; however, when disrupted, it directly compromises the structural integrity of the ventricular myocardium and renal parenchyma, ultimately impairing cardiac and renal functions (108, 109).

Elevated levels of glucose, UA, and FFAs can induce mitochondrial dysfunction, which leads to ROS accumulation and exacerbates mitochondrial dynamic disturbances and mitochondrial DNA damage, forming a vicious cycle (110–114). High glucose levels can promote excessive mitochondrial fission in renal podocytes, leading to glomerular damage and renal dysfunction (115). Impairment of the mitochondrial antioxidant defense system, together with increased mitochondrial ROS production, can harm renal cell membrane lipids, proteins, and DNA, thereby inducing podocyte apoptosis and endothelial cell damage, which are common features of acute and chronic kidney injuries (116, 117). Moreover, mitochondrial dynamic imbalance contributes to the pathogenesis of various CVDs. Mitochondrial dynamic homeostasis is essential not only for the growth, apoptosis, and migration of vascular endothelial and smooth muscle cells but also for the regulation of matrix metalloproteinase production by monocytes and macrophages, as well as extracellular matrix degradation, which are important initiating factors for vascular remodeling (118–122). Abnormal mitochondrial dynamics can impair vascular cell function and accelerate the onset and progression of vascular remodeling diseases such as atherosclerosis (118, 121). Additionally, hyperglycemia can promote time-dependent mitochondrial dysfunction in cardiomyocytes, potentially leading to diabetic cardiomyopathy (123). Given the important role of mitochondrial function in regulating cardiorenal injury, maintaining mitochondrial homeostasis during metabolic abnormalities is crucial.

### 4.6 Lipotoxicity and insulin resistance

In lipotoxicity during lipid metabolism disorders, FFA concentrations or intracellular lipid levels exceeds the storage capacity of adipose tissue and the oxidative capacity of various tissues for FFAs; consequently, lipid levels are abnormally elevated in the blood or are excessively deposited in nonadipose tissues, causing damage and toxicity to tissues and organs (124, 125). Lipotoxicity has been reported to increase CKD risk and considered as an independent risk factor for adverse cardiovascular events (126). It also increases the triglyceride and low-density lipoprotein cholesterol (LDL-C) levels; the deposition of these lipid components in the vascular wall leads to plaque formation, which causes vascular stenosis and obstruction, ultimately increasing the risk of atherosclerotic CVD (127). Lipotoxicity-induced liver inflammation may also lead to an imbalance in coagulation and fibrinolysis, making the blood hypercoagulable (128); consequently, cardiac and renal blood vessels develop thrombosis, which further promotes the onset and progression of cardiorenal diseases. Abnormal lipid metabolism also results in excessive FFA accumulation in nonadipose organs such as the heart and kidneys. When renal sinus fat abnormally accumulates, mechanical compression and inflammatory factor release can occur, triggering local hypoxia, oxidative stress, and fibrosis; consequently, nephron function is impaired (129, 130). Similarly, abnormal FFA buildup in cardiomyocytes activates ROS production, induces ER stress, and disrupts mitochondrial β-oxidation, leading to cardiomyocyte apoptosis, interstitial fibrosis, and contractile function impairment; ultimately, lipotoxic cardiomyopathy develops (131, 132). Early prevention and control of lipotoxicity are essential to effectively maintain cardiorenal function.

Insulin resistance is a core feature of metabolic disorders and is crucial in CVD and CKD development by promoting myocardial fibrosis, endothelial dysfunction, and lipid metabolism disorders (133, 134). Insulin resistance can activate the mTOR/S6K1 signaling pathway, affecting the expression of insulin receptors in cardiac and renal tissues, reducing insulin sensitivity, and ultimately damaging such tissues (135–137). Pulakat et al. found that the mTOR/S6K1 signaling pathway was activated in the cardiovascular tissues of rodent models with nutrient excess; this activation is associated with weakened insulin metabolic signaling, impairing NO-mediated vasodilation, causing cardiac diastolic dysfunction, and promoting renal tubulointerstitial fibrosis (138). Insulin resistance can also contribute to peripheral microvascular and skeletal muscle dysfunction (139, 140), which are linked to increased HF risk (141, 142). A positive correlation was found between insulin resistance and HF risk in older adults with diabetes (143). Overall, insulin resistance directly or indirectly contributes to myocardial and renal tissue damage, and mitigating it may help alleviate its negative effects on the cardiovascular system and kidneys.

## 5 Therapeutic options

**Figure 3:**
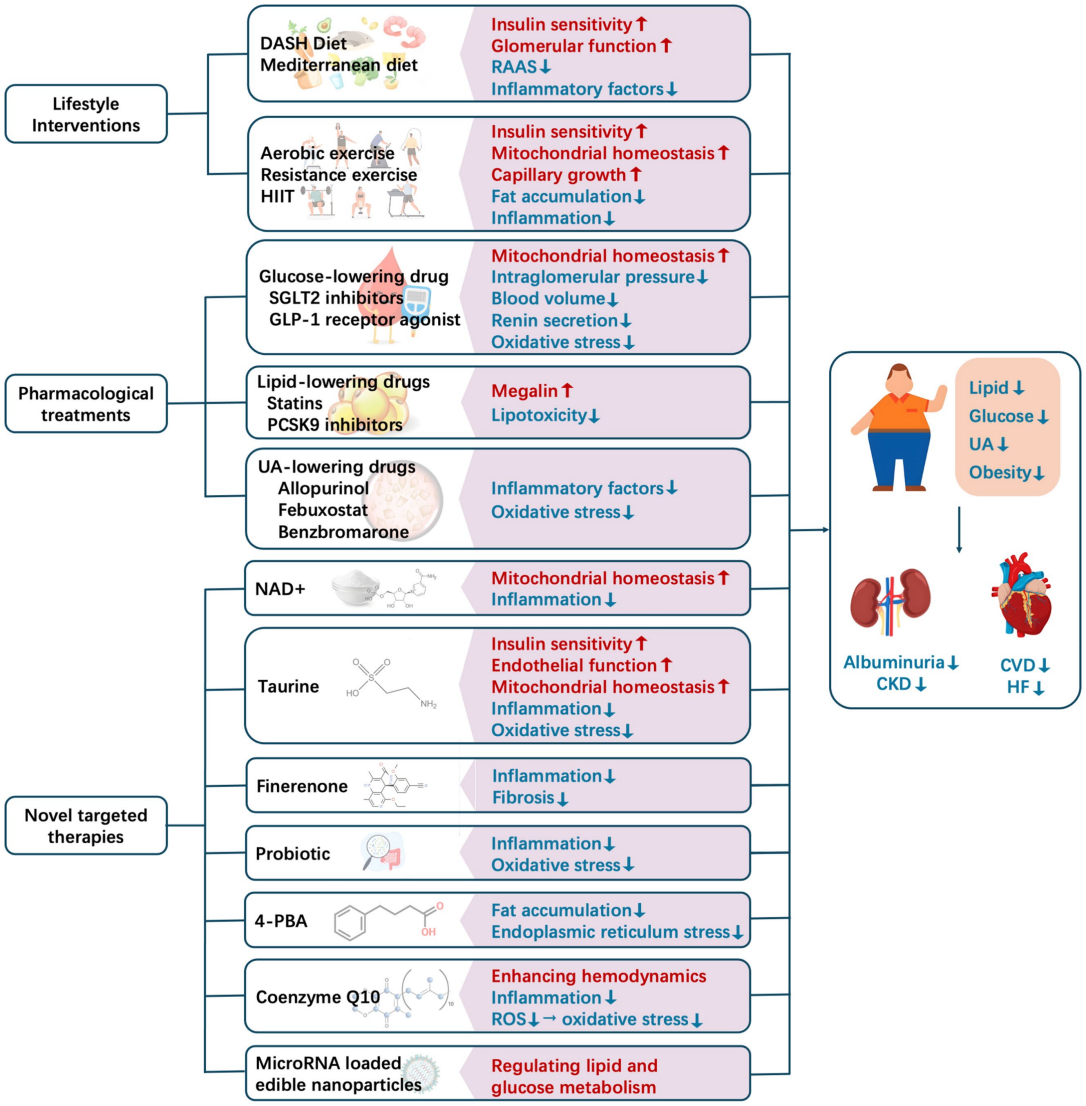
Current methods for intervening in and regulating metabolic conditions to improve cardiorenal outcomes. CKD, chronic kidney disease; CVD, cardiovascular disease; DASH, Dietary Approach to Stop Hypertension; HF, heart failure; HIIT, high-intensity interval training; NAD+, nicotinamide adenine nucleotide; RAAS, renin–angiotensin–aldosterone system; ROS, reactive oxygen species; UA, uric acid.

In this review, the analyses of observational studies and randomized trials have demonstrated that early metabolic regulation and subsequent albuminuria remission can accurately predict long-term improvements in cardiorenal clinical outcomes (144, 145). Metabolic conditions are not only a potent predictor of cardiorenal risk but also a modifiable therapeutic target. The following discussion focuses on the beneficial effects of metabolic regulation on cardiorenal diseases, with an emphasis on lifestyle interventions, pharmacological treatments, and novel targeted therapies (Figure 3).

### 5.1 Lifestyle interventions

Lifestyle interventions, including dietary modification and exercise, are fundamental to the management of metabolic abnormalities. By improving energy metabolism balance, reducing oxidative stress, and mitigating inflammatory responses, they offer multidimensional protection to the renal and cardiovascular systems. The Dietary Approach to Stop Hypertension diet emphasizes a high intake of vegetables, fruits, whole grains, and low-fat dairy products while limiting saturated fat and cholesterol consumption (146). Mechanistically, its high levels of potassium, magnesium, and dietary fiber enhance insulin sensitivity and inhibit RAAS activation (147, 148), thereby alleviating glomerular hyperfiltration. The Mediterranean diet emphasizes the intake of monounsaturated fatty acids (MUFAs), primarily from olive oil, fish, and nuts, to lower the inflammatory factor levels (149, 150). The CORDIOPREV study, which enrolled 1,002 patients with coronary heart disease, revealed that the crude incidence rate of major cardiovascular events per 1,000 person-years was 28.1 (95% CI, 27.9–28.3) in the Mediterranean diet group, which was significantly lower than 37.7 (95% CI, 37.5–37.9) in the low-fat diet group, with a log-rank *p*-value of 0.039 (151). The multivariate-adjusted HRs across the different models ranged from 0.719 (95% CI, 0.541–0.957) to 0.753 (95% CI, 0.568–0.998), indicating that the Mediterranean diet has cardiovascular protective effects (151). Furthermore, each one-point increase in the Mediterranean Diet Scale was associated with a 10% reduction in CKD risk (OR, 0.901; 95% CI, 0.868–0.935) (152), possibly because of MUFAs, which inhibit the TLR4/NF-κB pathway and reduce glomerular endothelial cell damage (153, 154).

Various types of exercise, including aerobic exercise, resistance exercise, and comprehensive exercise training, can effectively prevent and significantly improve metabolic abnormalities. For example, moderate-intensity aerobic exercise (e.g., brisk walking and swimming) activates the AMPK/PGC-1α pathway, thereby enhancing mitochondrial biogenesis and insulin sensitivity in both cardiac and skeletal muscles (155–157). For patients with metabolic abnormalities characterized by muscle loss and fat accumulation, resistance exercise can enhance muscle glucose uptake and reduce ectopic fat (158, 159). Furthermore, through alternating short bursts of high-intensity exercise and low-intensity recovery periods, high-intensity interval training (HIIT) significantly enhances mitochondrial oxidative phosphorylation efficiency and capillary growth (160, 161), improving cardiac function (162). Additionally, HIIT benefits renal function by influencing kidney-specific mRNA expression of genes related to endogenous antioxidant enzyme activity (*Gpx1, Sod1*, and *Cat*) and inflammation (*Kim1* and *Tnfrsf1b*) (163, 164).

### 5.2 Pharmacological treatments

Drug regimens should be tailored to specific metabolic disorders (e.g., hyperglycemia, hyperlipidemia, and hyperuricemia) while providing renal and cardiovascular protection.

For instance, SGLT2 inhibitors promote urinary glucose excretion, reduce blood volume, and lower intraglomerular pressure by inhibiting SGLT2 in the proximal tubule (165, 166). The SGLT2 inhibitor empagliflozin decreased the risk of kidney disease progression or cardiovascular death by 28% (95% CI 0.64–0.82, *p* < 0.0001) in the EMPA-KIDNEY trial (167). This drug also significantly reduced the risk of adverse cardiovascular events and hospitalization for HF in patients with T2DM in the EMPA-REG OUTCOME (168). GLP-1 receptor agonist, a glucose-lowering drug, enhances glucose-dependent insulin secretion while inhibiting gastric emptying and appetite. A meta-analysis conducted by Kristensen et al. revealed that GLP-1 receptor agonist reduced the risks of MACE by 12% (95% CI, 0.82–0.94, *p* < 0.0001) and a broad composite kidney outcome by 17% (95% CI, 0.78–0.89; *p* < 0.0001), mainly resulting from the decreased excretion of urinary albumin (169). More importantly, both SGLT2 inhibitors and GLP-1 receptor agonists exert cardiorenal protection through multiple mechanisms, including the regulation of mitochondrial function, inhibition of renin secretion, and reduction of oxidative stress (170, 171). SGLT2 inhibitors enhance mitochondrial biogenesis and mitophagy by activating the AMPK-PGC-1α pathway, while directly suppressing renin release by restoring sodium delivery to the macula densa (170, 172). GLP-1 receptor agonists stabilize mitochondrial membrane potential and optimize energy metabolism via the cAMP-PKA signaling cascade; they also indirectly modulate the RAAS by regulating the sympathetic tone (170, 172). Furthermore, both drug classes could suppress ROS generation and regulate calcium homeostasis, thereby attenuating the unfolded protein response to block ER stress–driven apoptotic pathways (170, 171); ultimately, cytoprotective effects are exerted in the heart and kidneys.

Statins, which regulate lipids, inhibit oxidized LDL generation (173) and suppress macrophage infiltration, thereby delaying the progression of atherosclerosis and glomerulosclerosis (174, 175). Lipid-lowering drugs, particularly PCSK9 inhibitors, reduce the risk of cardiovascular events by lowering LDL-C and lipoprotein(a) levels (176, 177). PCSK9 exacerbates albuminuria by interacting with and downregulating megalin, a proximal tubule receptor essential for protein reabsorption in the kidneys (178, 179). In experimental models, inhibiting PCSK9 maintained megalin levels, reduced albuminuria, and improved renal disease phenotype (180).

The protective effects of UA-lowering therapy on cardiorenal diseases require further exploration, and pharmacological treatment for symptomatic hyperuricemia may hold greater significance (181–183). Allopurinol reduces UA production by inhibiting xanthine oxidase activity, thereby blocking the conversion of hypoxanthine and xanthine into UA. A prospective cohort study by Goicoechea et al. demonstrated that allopurinol treatment significantly lowered serum UA and C-reactive protein levels, increased eGFR, and slowed the progression of kidney disease. Compared with the control group, allopurinol treatment reduced the risk of cardiovascular events by 71% (*p* = 0.026) (184). Similarly, the composite renal event rate was significantly lower in the febuxostat group than in the placebo group (relative risk [RR], 0.68; 95% CI 0.46–0.99), with a notably higher eGFR (mean difference: 2.89 mL/min/1.73 m^2^; 95% CI 0.69–5.09) (185). However, in high-risk patients such as those with diabetes or CKD, the impact of febuxostat on cardiovascular risk, compared with allopurinol, remains unclear in previous studies, with potential effects ranging from neutral to either reduced or increased risk; the underlying mechanisms are still unclear (186–188). In addition, in salt-induced hypertensive rat models, benzbromarone significantly reduced advanced oxidation protein products and attenuated oxidative stress, suggesting its substantial potential for preventing CVD and CKD (189).

### 5.3 Novel targeted therapies

Novel medications targeting multiple pathways, including chronic inflammation, ER stress, endothelial function, and mitochondrial function, are warranted.

Recently, nicotinamide adenine nucleotide (NAD^+^) has been discovered to regulate immune function and inflammation (190, 191). Treatment with nicotinamide riboside (NR), an NAD^+^ intermediate, in T2DM mice prevented the increase in albuminuria, urinary kidney injury molecule-1 excretion, and renal pathological changes; such prevention was due to reduced inflammation, at least partially by inhibiting the activation of the cGAS/STING signaling pathway (192). Additionally, NR increased SIRT3 activity and improved mitochondrial function, thereby reducing mitochondrial DNA damage (192). Moreover, through its anti-inflammatory and antioxidant activities, taurine, a sulfur-containing amino acid, helps alleviate endothelial dysfunction caused by metabolic abnormalities, prevent mitochondrial dysfunction, and help regulate vascular pressure (193–195). In patients with T2DM, taurine supplementation significantly reduces insulin resistance, oxidative stress, inflammation, and endothelial markers (193). Furthermore, finerenone, as a novel nonsteroidal mineralocorticoid receptor antagonist, is known to reduce inflammation and fibrosis, thereby exerting cardiorenal protective effects (196).

A previous study reported the interconnection between gut microbiota, metabolic abnormalities, and chronic inflammation (197). Trimethylamine-N-oxide (TMAO), a metabolite of gut microbiota, activates the TLR4/NF-κB pathway, thereby exacerbating inflammation; it is also closely associated with atherosclerosis and renal pathological changes (198–200). Clinically, probiotic therapy has been shown to reduce TMAO levels in patients with unstable angina (201). In patients with DN, Dai et al. found that probiotics could improve glucose and lipid metabolism and reduce inflammation and oxidative stress, thereby delaying the progression of albuminuria and renal function impairment (202).

The chemical 4-PBA alleviates ER stress by stabilizing protein folding; it also reduces fat accumulation in zebrafish fed a high-fat diet (203, 204). Several animal experiments have demonstrated that 4-PBA can reduce tubular cell apoptosis and renal fibrosis (205). Moreover, exogenous 4-PBA supplementation can inhibit atrial fibrosis in mice with atrial fibrillation induced by a high-fat diet (206), prevent cardiac rupture and remodeling in mice with MI (207), and inhibit myocardial hypertrophy and interstitial fibrosis caused by pressure overload (208). Additionally, nutritional supplements such as coenzyme Q10 (CoQ10), a coenzyme in the mitochondrial respiratory chain, can reduce ROS production, thereby improving oxidative stress responses. Clinically, CoQ10 supplementation has beneficial effects on the lipid profile and helps lower blood pressure (209). It also helps prevent acute kidney injury in male diabetic rats, primarily by enhancing renal hemodynamics and reducing oxidative stress (210). Furthermore, CoQ10 can improve cardiovascular health, potentially by reducing inflammation and oxidative stress, thereby decreasing fibrosis (211). MicroRNAs (miRNAs) participate in the epigenetic regulation of genes involved in lipid and glucose metabolism, with some being dysregulated in metabolic and cardiorenal diseases (212–214). Research on miRNA-loaded edible nanoparticles offers promising new perspectives for clinical interventions targeting metabolic disorders and cardiorenal diseases (215).

Given that single-pathway interventions have limited efficacy, multi-pathway synergistic therapies have emerged as the core strategy for managing metabolic abnormalities. Regulating metabolism can effectively alleviate chronic inflammation, relieve ER stress, and optimize mitochondrial function, thereby improving cardiorenal outcomes. These mechanisms provide an important theoretical basis for the prevention and treatment of complications related to metabolic abnormalities.

## 6 Conclusion

**Figure 4:**
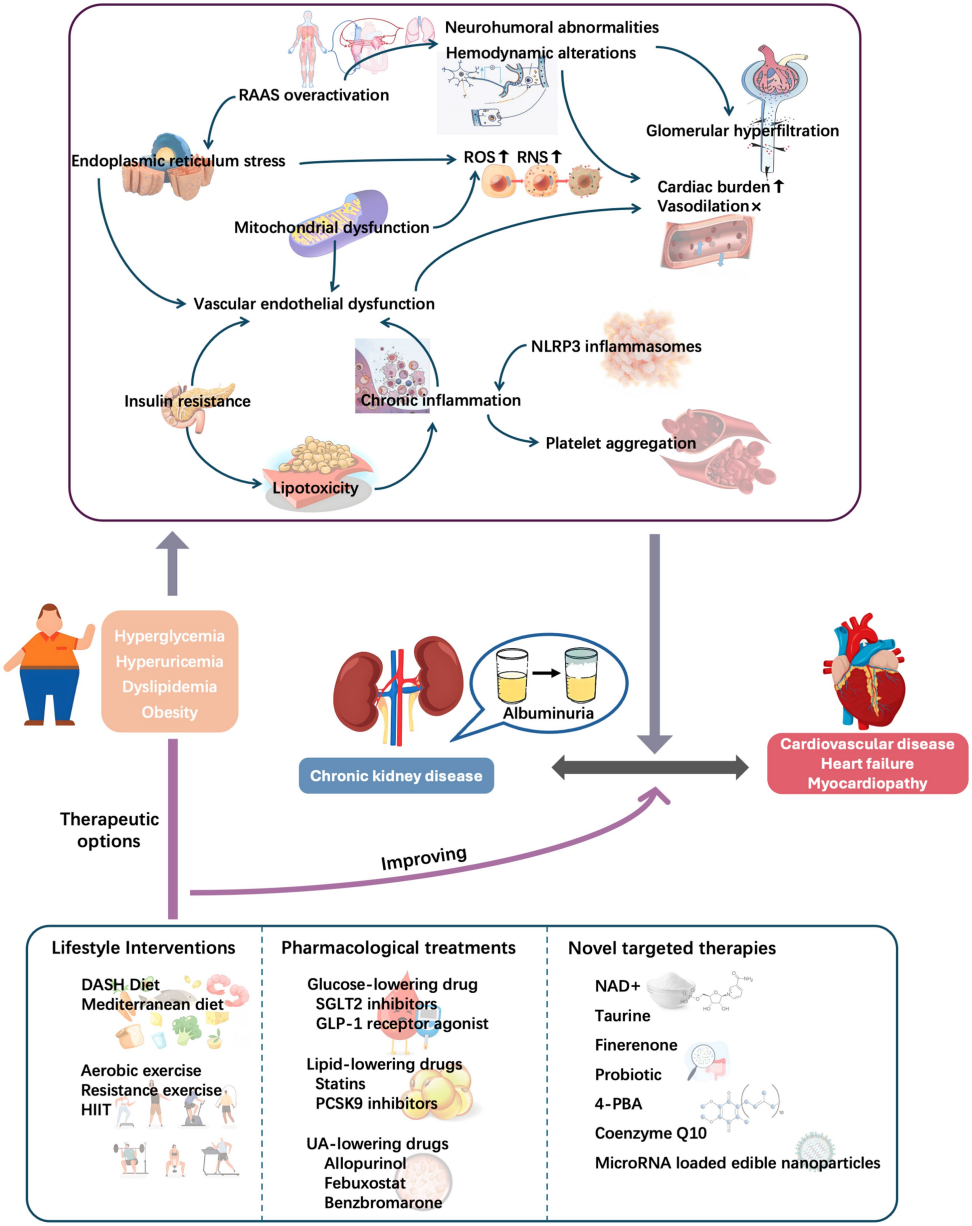
Role of metabolic conditions in cardiorenal diseases: Initiating pathways and therapeutic targeting. DASH, Dietary Approach to Stop Hypertension; HIIT, high-intensity interval training; NAD+, nicotinamide adenine nucleotide; NLRP3, nodular receptor protein 3; RAAS, renin–angiotensin–aldosterone system; RNS, reactive nitrogen species; ROS, reactive oxygen species; UA, uric acid.

Given the associations among metabolic conditions, albuminuria, and CVD (Figure 4), early screening, comprehensive management, and targeted therapies are important. Targeted intervention in metabolic abnormalities may effectively control albuminuria, delay CVD progression, and improve the overall prognosis of cardiorenal disease. Future research should further explore the underlying mechanisms so that more precise prevention and treatment strategies can be developed, providing patients with more comprehensive health protection.
